# The challenge of targeting metastasis

**DOI:** 10.1007/s10555-015-9586-9

**Published:** 2015-09-02

**Authors:** Isaiah J. Fidler, Margaret L. Kripke

**Affiliations:** Department of Cancer Biology, The University of Texas MD Anderson Cancer Center, 1515 Holcombe Boulevard, Unit 173, Houston, TX 77030 USA; Department of Immunology, The University of Texas MD Anderson Cancer Center, 1515 Holcombe Boulevard, Unit 173, Houston, TX 77030 USA

**Keywords:** Biologic heterogeneity, Genetic instability, Organ microenvironment, Metastasis progenitor cell

## Abstract

Metastases that are resistant to conventional therapy are the major cause of death from cancer. In most patients, metastasis has already occurred by the time of diagnosis. Thus, the prevention of metastasis is unlikely to be of therapeutic benefit. The biological heterogeneity of metastases presents a major obstacle to treatment. However, the growth and survival of metastases depend on interactions between tumor cells and host homeostatic mechanisms. Targeting these interactions, in addition to the tumor cells, can produce synergistic therapeutic effects against existing metastases.

## Introduction

Twenty years ago, a close family member was diagnosed at the age of 54 with colon cancer with extensive liver metastases. He was told by his family physician that he would probably not live another 6 months. After the successful removal of his primary tumor, he came to The University of Texas MD Anderson Cancer Center to explore any options available to treat his liver metastases. He was placed on a highly experimental protocol involving direct introduction of chemotherapeutic drugs into the liver. The metastases began to regress, and he returned to work. With the exception of a few bad days following the intermittent chemotherapy, his quality of life was excellent, and he was alive to enjoy the arrival of a second grandchild. But 3 years later, the metastases began to regrow, and when he asked about trying a different drug regimen, he was told, “I’m sorry, but there isn’t another option.” He returned to his home and died there within a few months at the age of 57.

Unfortunately, 20 years later, this outcome is still not unique. This year alone nearly 590,000 people in the USA will die from cancer [1]. In spite of the promise of new therapies targeting genes gone awry in cancer cells, the fact remains that even with these more effective, less toxic approaches, patients still die from metastases that eventually escape control. In his book, *The Emperor of All Maladies*, Dr. S. Mukherjee concludes that the only way to deal with this problem using current approaches is to ensure that drug development stays one step ahead of the emergence of resistant metastases [2]. Consequently, many recent studies are directed toward prevention of metastasis. However, this approach is also not likely to change the outcome of cancer because in many patients, by the time of diagnosis, metastasis has already occurred [3]. If this were not the case, surgical excision of primary neoplasms would be curative.

## The metastatic process

Metastasis, the transfer of disease from one organ or part to another not directly connected to it, remains the primary challenge in treating cancer [3, 4]. Although metastasis can occur when primary tumors are small, in most cases, metastasis is associated with large primary neoplasms. This is supported by data indicating that surgical excision of small lesions is often curative and forms the basis for tumor, nodes, metastasis (TNM) staging [4]. In advanced disease, metastasis has occurred and lesions are established in distant organs, so targeting early steps in the metastatic process may not be relevant to therapy. Targeting metastasis must therefore distinguish between prevention of the process and therapy of existing metastatic lesions.

The pathogenesis of cancer metastasis consists of a series of sequential and interrelated steps, each of which can be rate limiting. Failure to complete any of the steps can prevent the formation of secondary lesions. After the initial transformation and growth of cells, vascularization must occur if a tumor mass is to exceed 1 mm in diameter [5, 6]. The synthesis and secretion of several proangiogenic factors by tumor and host cells and the absence of antiangiogenic factors play a key role in establishing a capillary network from the surrounding host tissue [7, 8]. Next, local invasion of the host stroma occurs as a consequence of the enhanced expression of a series of enzymes (*e.g.*, collagenase) [9–11]. Once the invading cells penetrate the lymphatic or vascular channels, they may grow there, or a single cell or clumps of cells may detach and be transported within the circulatory system. The tumor emboli must survive immune defenses and the turbulence of the circulation [3, 4]. They must then arrest in the capillary bed of distant organs, extravasate into the organ parenchyma, and proliferate to establish a micrometastasis. The growth of these microscopic lesions requires the development of a vascular supply and evasion of host immune cells [3, 4].

Only a few cells in a primary tumor are able to give rise to a metastasis [12, 13], and tumor cells that fail to complete all of the steps of the process are eliminated [12]. The entrance of tumor cells into the circulation is common, and more than a million cells per gram of primary tumor can be shed daily [14]. Circulating tumor cells have been found in the blood of many cancer patients [15], whether their presence can predict that metastasis will occur is still unclear [16, 17]. For example, circulating tumor cells were found in the blood of patients with benign colon diseases [16]. Also, the use of peritoneovenous shunts to reduce ascites in patients with advanced ovarian cancer [17] allowed millions of tumor cells to enter the circulation every 24 h. These patients rarely developed secondary lesions [17]. Even the arrest in capillary beds of various organs does not predict the subsequent development of metastases. Quantitative experiments using radioactive-labeled melanoma cells injected intravenously into syngeneic mice demonstrated that only <0.1 % of the injected cells survived to produce metastases. Moreover, although the tumor cells reached all organs, metastases developed in only a few [12]. Thus, the presence of tumor cells in the circulation does not necessarily predict that metastasis will occur [18].

Oncologists are well aware that metastasis is not a random process because the pattern of metastasis produced by different cancers is predictable [3, 4]. This conclusion was first enunciated by Stephen Paget in 1889 when he published the report, “Distribution of Secondary Growths in Cancer of the Breast,” to answer the question, “What is it that decides what organs shall suffer in a case of disseminated cancer?” [19]. Paget studied the autopsy records of 735 women with breast cancer, questioning the discrepancy between the relative blood supply and the frequency of metastasis in several organs. He noticed the high incidence of metastasis in the lung, liver, ovary, and specific bones and the low incidence in the spleen. His findings contradicted the prevailing theory proposed by Virchow [20] that metastasis can be explained by the simple lodgment of tumor cell emboli in the proximal vascular bed. Paget concluded that “remote organs cannot be altogether passive or indifferent regarding embolism” and proposed the “seed and soil” principle stating “When a plant goes to seed, its seeds are carried in all directions; but they can only live and grow if they fall on congenial soil.” Paget ended with the statement, “All reasoning from statistics is liable to many errors. But the analogy from other diseases seems to support what these records have suggested, the dependence of the seed upon the soil. It is our opinion that the best work in the pathology of cancer is now done by those who, like Mr. Balance and Mr. Shattock, are studying the nature of the seed. They are like scientific botanists, and he who turns over the records of cases of cancer is only the ploughman, but his observations of the properties of the soil may also be helpful” [19].

The “seed and soil” concept lay dormant until studies were performed in which fragments of lung, ovary, and kidney tissues were grafted into the subcutaneous space or muscle of syngeneic mice, following which radiolabeled melanoma cells were injected intravenously [21]. Although tumor cells reached the vasculature of all organs, metastases developed only in the orthotopic and grafted lungs and ovaries, but not in the orthotopic or grafted kidneys [21], demonstrating the essential contribution of the “soil” to this process. The “seed and soil” hypothesis is now widely accepted [22]. The “seed” has been renamed as the initiating cell, progenitor cell, cancer stem cell, or metastatic cell, and the “soil” is named the stroma, host factors, niche, or organ/tissue microenvironment. Regardless of the terminology, it is clear that the development and survival of metastases are dependent on the continuous interplay between tumor cells with unique metastatic properties that preexists in the parental tumor [22, 23] and the microenvironment of specific receptive organs [22–26]. Thus, the establishment of a metastasis consists of two components: the selected metastatic cell and the unique organ microenvironment [27, 28].

## Strategies to prevent metastasis

A current focus of cancer research is the development of agents that target specific genetic alterations in primary tumors. This is an admirable goal because it promises to produce less toxic therapies tailored to individual cancers. However, the results to date have been less beneficial than expected for a variety of reasons [29–32]. Chief among these is the biological heterogeneity of primary tumors and metastases [33–37] and the plasticity of cancer cells [29–31] due to genetic instability [4, 35]. Primary neoplasms consist of multiple subpopulations of cells with heterogeneity in diverse properties, such as gene expression, karyotype, growth rate, antigenicity/immunogenicity, cell surface receptors, marker enzymes, sensitivity to different cytotoxic drugs, invasion, and metastasis [3, 4, 34]. The cellular composition of metastases is also heterogeneous [38, 39], both within a single metastasis (intralesional heterogeneity) and among different metastases (interlesional heterogeneity) [40]. Karyotypic analyses [41–45] and single-cell sequencing [46] demonstrated that metastases are clonal in origin and can develop from a single surviving cell [43]. Their most daunting characteristic, however, is their increased genetic instability [47]. This property enables new variants to arise rapidly; those that are resistant to therapy have a selective advantage and eventually overgrow the sensitive cells, leading to the reemergence of metastatic disease [47].

Basing therapy on the identification of genes uniquely expressed in a primary tumor may be of limited value because of the heterogeneity in gene expression among different zones within a single primary tumor [37, 40, 41], differences in gene expression between cells in a primary tumor and those populating distant metastases, or differences among different metastases arising in the same or different organs [3, 4, 48–50]. Thus, the genomic instability of tumor cells in general and the increased genomic instability of metastatic cells in particular may render the identification of a reliable, genetically based target for therapy impractical. In fact, to date, targeting such genes has not yet led to durable responses in most patients.

Whereas targeting one or several steps in the process of metastasis may prevent the establishment of distant lesions, in most patients, by the time of diagnosis, metastasis has already occurred [3, 4]. If this were not the case, surgical resection of primary tumors would be curative. Many current studies are attempting to inhibit various steps of the metastatic process. For example, to enter the circulation, tumor cells must invade the host stroma. This invasion occurs by multiple mechanisms including the release of matrix metalloproteinases. Inhibition of these enzymes can inhibit invasion and, therefore, metastasis [10]. Embolization of circulating tumor cells is enhanced by clumping with platelets [51–54]. Administration of aspirin to mice can prevent this interaction and thus decrease the establishment of experimental metastasis, but it does not reduce preexisting lesions [53, 54].

In 1971, Folkman advanced the concept of “tumor angiogenesis: therapeutic implications” [6]. Angiogenesis, defined as the “generation of new blood vessels,” is essential for the growth of tumor mass beyond 1 mm in diameter [6, 7]. Inhibition of angiogenesis, therefore, was envisioned to prevent the growth of metastases beyond the 1-mm size, *i.e.*, prevent metastases from reaching the size that can be diagnosed in patients and be clinically relevant.

Numerous studies directed at inhibiting angiogenesis for therapy of metastasis have now been published [7, 55, 56]. The clinical efficacy of antiangiogenesis therapy that targets VEGF or its receptors has been disappointing [56]. In some experimental systems, anti-VEGF therapy actually led to accelerated formation of metastasis [57]. In a recent study with small cell lung cancer and pancreatic ductal adenocarcinoma, the administration of low-dose cilengitide and verapamil increased angiogenesis of primary tumors, but it also increased the potency of gemcitabine, leading to reduced tumor growth and spread [58]. These seemingly contradictory results can readily be explained by the definition of “angiogenesis,” *i.e.*, the creation of new blood vessels. In most experimental systems, the injection of tumor cells into ectopic or orthotopic organs [26] is followed by rapid growth of tumors that requires the establishment of new vasculature [59]. In clinical trials, however, the challenge is to treat tumors that failed to respond to conventional therapy [60]. These tumors represent chronic lesions with a well-established blood supply [4]. Preventing the development of new vasculature is irrelevant and unlikely to lead to regression of the lesions. Metastasis can develop in organs, such as the lung and brain, that have abundant capillary beds. Tumor cells can often proliferate around existing vessels in a phenomenon labeled as vessel co-option [61, 62]. These preexisting blood vessels negate the need for generating new vasculature, and thus, antiangiogenic therapy is unlikely to be effective.

Clearly, it is not enough to prevent metastases since these mainly arise prior to surgical resection of the primary tumor. At best, such approaches can only prevent the development of secondary metastases, which are derived from metastatic lesions. Therefore, the major challenge is to treat existing metastases, not prevent new metastases.

## Targeting established metastases

The survival and growth of metastatic foci is dependent on multiple continuous interactions with host homeostatic mechanisms. The conclusion by Dvorak [63] and later by Riss *et al.* [64] that “cancer is a wound that does not heal” is based on the finding that inflammatory and repair processes can aid tumor growth and, hence, be targeted for therapy.

As stated above, targeting the existing tumor vasculature rather than preventing angiogenesis should be a major focus of research [47–49]. One approach to accomplish this has been termed “the normalization of blood vessels,” defined as the pruning of blood vessels within neoplasms and reduction of increased vessel permeability [65–67]. The administration of humanized IgG_L_ monoclonal antibody (bevacizumab) with high affinity for isoforms of VEGF-A in combination with chemotherapy to treat established neoplasms was proposed to achieve this goal [68]. In other systems, administration of bevacizumab to induce normalization of blood vessels in xenografted ovarian carcinoma and esophageal cancer monitored by increased pericytes attached to endothelial cells decreased the uptake of antibody by the tumors [69]. A similar decrease in the delivery of chemotherapeutic drugs to tumors following anti-VEGF therapy has also been reported [70]. Targeting vascular pericytes in established hypoxic tumors increased angiopoietin-2 expression and led to increased lung metastases. Depletion of pericytes and targeting of angiopoietin-2 signaling restored vascular stability and decreased tumor growth and production of metastasis [71]. Regardless of these contrasting data, the value of normalizing tumor blood vessels for the treatment of metastases in some organs may have beneficial results and should be tested. Inhibition of angiogenesis can lead to accelerated formation of metastasis [57] which, again, emphasizes the importance of distinguishing between targeting angiogenesis in primary neoplasms and in metastases. The latter are the challenge for the treatment of patients [55] in systems where metastases are well established and, thus, represent the clinical reality. Moreover, in organs such as the brain and lung where vessel co-option is common [62, 63], using antiangiogenic therapy may not be effective.

The production of growth factors by tumor cells, and more so by host cells, can accelerate expansion of tumors. Targeting growth factors, such as EGF or the EGF receptors, have produced significant therapeutic effects [72, 73]. The influence of host inflammatory cells on the growth and spread of cancer is also well documented [74–76]. Targeting these inflammatory changes has been shown to have additive therapeutic effects when combined with chemotherapeutic agents that target the tumor cells [77]. Pancreatic carcinoma is associated with marked fibrosis and stromal microfibroblasts. This stroma has been shown to promote the growth of pancreatic cancer [77, 78], and therefore, targeting the stroma is likely to have therapeutic benefits in this highly malignant disease which responds poorly to chemotherapy directed against the tumor cells.

In some organs, activation of immune cells such as lymphocytes [79, 80] can produce dramatic therapeutic effects. Macrophages can discriminate between normal and tumorigenic cells [81], and activating macrophages *in vivo* by intravenous injection of liposomes containing MTP-PE has been shown to cure osteogenic sarcoma lung metastases in children [82, 83].

The diffusion distance of oxygen from capillaries within tissues is about 150–200 μM [84, 85]. Immunohistochemical analysis revealed that tumor cells located less than 100 μM from a capillary in brain metastasis are viable, whereas more distant tumor cells undergo apoptosis [59]. This study also demonstrated that the blood-brain barrier (BBB) is intact in and around brain metastases smaller than 0.25 mm in diameter but is leaky in larger metastases [59]. The progressive growth of tumor cells in experimental brain metastasis and brain tumors depends on the expression of VEGF/vascular permeability factor (VPF) [86]. Transfection of human lung cancer cells with an antisense VEGF-165 gene decreased the formation of brain metastasis. In contrast, transfection of cells from human squamous carcinoma of the lung with sense VEGF-121 or sense VEGF-165 neither increased nor inhibited the formation of brain metastasis. These results suggest that while VEGF expression is necessary, it is not sufficient for the formation of brain metastasis and that inhibition of VEGF presents an important target for therapy of brain metastasis [86].

Targeting the established vasculature of metastases should provide a susceptible target for therapy. Inhibiting phosphorylation of platelet-derived growth factor receptors on endothelial cells by using imatinib combined with taxol has been reported to kill endothelial cells in vessels of multidrug-resistant prostate cancer leading to a decrease in incidence and size of experimental bone metastases [87]. Similarly, inhibition of the epidermal growth factor receptor and VEGFR phosphorylation on tumor-associated endothelial cells can produce apoptosis in tumor vasculature and tumor cells of orthotopic human colon carcinoma in nude mice [88]. Induction of apoptosis in tumor-associated endothelial cells leads to therapy of orthotopic human pancreatic carcinoma in nude mice [89]. Macitentan, a dual endothelin receptor antagonist, combined with taxol produced a significant therapy of established multidrug-resistant orthotopic human ovarian carcinoma growing in nude mice [90]. This combination produced apoptosis of tumor-associated endothelial cells followed by apoptosis of tumor cells deprived of oxygen [90].

The organ microenvironment is conducive to the development, progression, and survival of brain metastases. Metastatic cells in the brain and primary brain tumors are resistant to chemotherapy. Since the growth of tumor lesions in the brain exceeding 0.25 mm in diameter is associated with expression of VEGF/VPF [59] and leakiness of the blood-brain barrier, the metastatic cells in the brain as well as cells in primary brain tumors are resistant to chemotherapy [91]. A recent work from our laboratory reported that in clinical cases of brain metastases and experimental models, tumor cells are surrounded by activated GFAP-positive astrocytes. Astrocytes normally establish gap-channel junctions with endothelial cells and neurons; however, such junctions also occur between astrocytes and tumor cells [92, 93]. One physiological function of astrocytes is to protect neurons from damage by toxic substances. However, tumor cells also benefit from this mechanism. Astrocytes release endothelin-1 which binds to endothelin receptors in tumor cells [94]. The activation of the endothelin receptors leads to increased expression of survival genes and proteins and, hence, increased resistance to chemotherapeutic drugs. Antagonists of the endothelin axis, combined with chemotherapy, prevent the production of antiapoptotic proteins (GSTA5, Bcl_2_L_1_, TWIST1) in tumor cells and increase their vulnerability to anticycling drugs [94].

Astrocytes have also been implicated in the establishment and growth of melanoma brain metastasis by secretion of IL-23, which induces neuroinflammatory changes and repair [95].

## Conclusion

Targeted therapy requires a target. Focusing only on the tumor cell (seed) as an approach to cancer therapy misses the opportunity to also manipulate the host microenvironment (soil) to render it less hospitable for the growth and survival of metastases. Although the metastatic cell is an elusive target because of its genetic instability, the organ microenvironment represents a stable target that might be manipulated to the detriment of even established metastases. Treatment of metastases should take advantage of the dependence of metastatic tumor cells on a receptive organ microenvironment. Simultaneously targeting tumor cells and manipulating the organ microenvironment are more likely to produce the long-awaited therapy of metastasis than focusing solely on the metastatic cells.
